# Parallel gene expression evolution in natural and laboratory evolved populations

**DOI:** 10.1111/mec.15649

**Published:** 2020-10-12

**Authors:** Sheng‐Kai Hsu, Chaimae Belmouaden, Viola Nolte, Christian Schlötterer

**Affiliations:** ^1^ Institut für Populationsgenetik Vetmeduni Vienna Vienna Austria; ^2^ Vienna Graduate School of Population Genetics Vetmeduni Vienna Vienna Austria; ^3^ Present address: Faculty of Fundamental and Applied Sciences of Poitiers France

**Keywords:** *Drosophila melanogaster*, experimental evolution, gene expression, temperature adaptation

## Abstract

Ecological adaptation is frequently inferred by the comparison of natural populations from different environments. Nevertheless, inference of the selective forces suffers the challenge that many environmental factors covary. With well‐controlled environmental conditions, experimental evolution provides a powerful approach to complement the analysis of natural populations. On the other hand, it is apparent that laboratory conditions differ in many ways from natural environments, which raises the question as to what extent selection responses in experimental evolution studies can inform us about adaptation processes in the wild. In this study, we compared the expression profiles of replicated *Drosophila melanogaster* populations which have been exposed to two distinct temperature regimes (18/28 and 10/20°C) in the laboratory for more than 80 generations. Using gene‐wise differential expression analysis and co‐expression network analysis, we identified 541 genes and three coregulated gene modules that evolved in the same direction in both temperature regimes, and most of these changes probably reflect an adaptation to the space constraint or diurnal temperature fluctuation that is common in both selection regimes. In total, 203 genes and seven modules evolved temperature‐specific expression changes. Remarkably, we detected a significant overlap of these temperature‐adaptive genes/modules from experimental evolution with temperature‐adaptive genes inferred from natural *Drosophila* populations covering two different temperature clines. We conclude that well‐designed experimental evolution studies are a powerful tool to dissect evolutionary responses.

## Abstract 2

see also the Perspective by Mark A. Phillips and Molly K. Burke.

## 1 INTRODUCTION

Reverse ecology, where genomic tools are used to study ecology without *a priori* knowledge of the phenotypic characteristics of the studied populations, has become a highly popular approach to study the genetic basis of local adaptation in natural populations. Contrasting individuals/populations from two different environments, such as heavy metal‐polluted soil versus uncontaminated soil (Turner et al., 2010), northern versus southern populations (Zhao et al., 2015), and marine versus freshwater populations (Lamichhaney et al., 2012), has been widely applied to unravel the genetic or phenotypic differentiation contributing to local adaptation. Rather than focusing on two extreme environments, it is also possible to compare populations along a geographical cline (e.g., latitude, altitude) where multiple environmental factors vary (Huey, 2000; Lankinen, 1993; Porcelli et al., 2016; Romero Navarro et al., 2017; Stinchcombe et al., 2004). Despite the undoubted success of reverse ecology, an important limitation is that typically more than one environmental factor differs among the groups compared. Hence, even when an unambiguous selection response is detected, the connection to the causative ecological factor remains correlative.

Evolve and re‐sequence (E&R) (Long et al., 2015; Schlötterer et al., 2015), which combines experimental evolution with whole genome sequencing, provides an alternative approach to study the genetic basis of adaptive traits. The advantage of E&R is not only a controlled environment, but also the possibility to follow the evolution of replicate populations across many generations. Nevertheless, while E&R has been very successful in demonstrating strong selective responses, only very rarely could the causative genes be identified (e.g., Martins et al., 2014). This is probably is the result of a large number of selection targets in combination with few generations and small population sizes. Beyond the limited mapping resolution of E&R studies, experimental evolution faces a conceptual challenge (Hoffmann et al., 2001). The environment of the experimental populations is dramatically different from natural environments, resulting in considerable adaptation to laboratory conditions. The biggest, but largely untested concern comes from the simplicity of the laboratory environment. Pleiotropic effects of genes responding to selection may have different consequences in natural and laboratory populations. In the simple, rather unconstrained laboratory environment, selection responses may be realized that cannot occur in the wild because of pleiotropic effects. Hence, the question arises as to what extent selection responses in E&R studies can inform us about adaptation processes in the wild rather than to laboratory‐specific conditions.

Because temperature is one of the most important environmental variables imposing selection pressure on natural populations, we studied the adaptive response of two *Drosophila melanogaster* populations that were exposed to hot and cold temperature regimes (Orozco‐terWengel et al., 2012; Tobler et al., 2014). With temperature adaptation being a highly complex phenotype with a polygenic basis (Barghi et al., 2019; Hoffmann & Hercus, 2000; Hoffmann et al., 1995), we focused on gene expression changes to determine the selection response because more consistent changes are expected for these molecular phenotypes than for genetic changes (Barghi et al., 2020). We distinguished putative temperature‐adaptive genes and coregulated modules with expression changing in opposite directions in hot and cold temperature regimes from those with expression changes shared between the two regimes. Based on gene ontology (GO) enrichment analysis, we inferred the potential functional requirements for the divergent and parallel adaptation. We also provided evidence for temperature adaptation in the laboratory mimicking the evolution in natural *Drosophila* populations covering two different temperature clines. We conclude that well‐designed experimental evolution studies are a powerful tool to dissect the evolutionary response to different environmental factors.

## 2 MATERIALS AND METHODS

### 2.1 Experimental evolution

**Figure 1:**
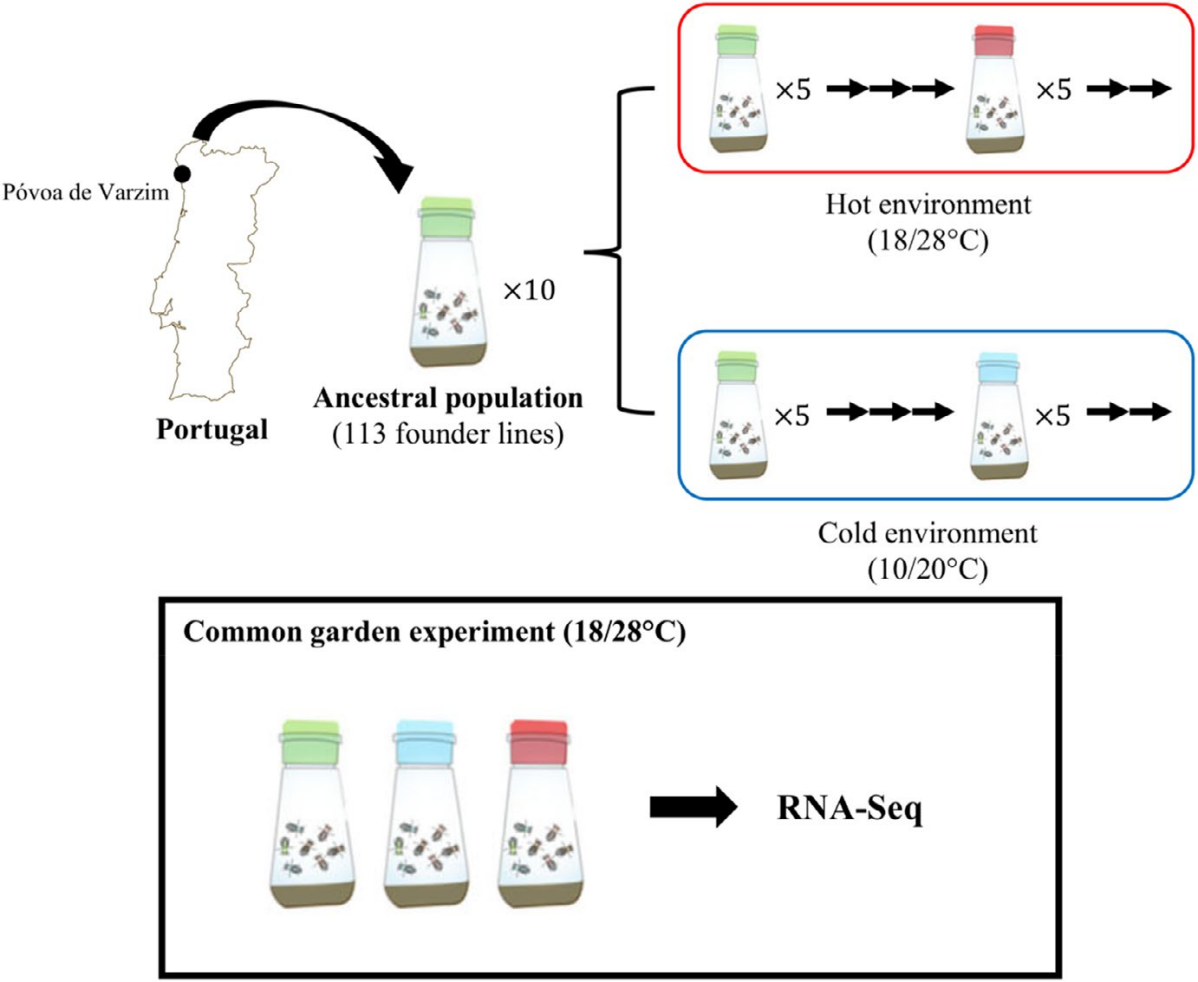
Experimental design. In total, 113 isofemale lines derived from a natural *Drosophila melanogaster* population (from Póvoa de Varzim, Portugal) were used to generate 10 independent replicated populations by pooling five females from each isofemale line. Five replicates were maintained at a high temperature regime at 18/28°C°C under a 12‐hr dark/12‐hr light circadian cycle (hot‐evolved replicates) while the other five replicates were maintained at a low temperature regime at 10/20°C°C under a 12‐hr dark/12‐hr light circadian cycle (cold‐evolved replicates). The census population size is between 1,000 and 1,250 adults per generation. At generation 155 and 81 of hot‐ and cold‐evolved replicates, respectively, a common garden experiment was performed at hot environment for two generations to collect for RNA‐Seq samples [Colour figure can be viewed at wileyonlinelibrary.com]

The design of this experimental evolution study has been previously described (Orozco‐terWengel et al., 2012; Tobler et al., 2014). Briefly, 113 isofemale lines were derived from a natural *Drosophila melanogaster* population collected in northern Portugal in summer 2008. These isofemale were kept in the laboratory at 18°C for five generations before constituting the ancestral population of the evolution experiments. Ten independent replicated populations were generated by pooling five females from each isofemale line. Five replicates were maintained at a high temperature regime at 28/18°C under a 12‐hr light/12‐hr dark circadian cycle (hot‐evolved replicates) while the other five replicates were maintained at a low temperature regime at 20/10°C under a 12‐hr light/12‐hr dark circadian cycle (cold‐evolved replicates). The census population size is between 1,000 and 1,250 adults per generation (Figure 1). Since the setup of the experimental evolution, all the isofemale lines have been reared at 18°C at a small population size (~50 adult flies per vial) to allow a reconstitution of the ancestral population (Nouhaud et al., 2016).

### 2.2 Common garden experiment

To avoid environmental or transgenerational effects on gene expression profiles of different evolved replicates and the ancestral populations, we performed a common garden experiment for two generations before sampling for RNA sequencing (RNA‐Seq). The common garden was set up when the hot‐evolved replicates reached 155 generations and cold‐evolved replicates reached 81 generations. Five replicates of reconstituted ancestral populations were generated by pooling five females from each of the isofemale lines which seeded the evolution experiment (Nouhaud et al., 2016). Five replicates of each evolved population (cold and hot) and reconstituted ancestral populations were reared for two generations under common garden conditions under the hot temperature regime (daily fluctuating 28/18°C environment) with density control (400 eggs per bottle). At the second generation, freshly eclosed adult flies were randomly mated for 3 days, sexes were separated afterwards under CO_2_ anaesthesia and aged for 2 days in a vial containing up to 50 flies. Fifty males of each replicate were flash‐frozen in liquid nitrogen at 4 p.m. at the age of 5 days and stored at −80°C until RNA extraction. We focused on male flies because a previous study detected allometric changes during adaptation for females, which were much weaker for males (Hsu et al., 2020).

### 2.3 RNA‐Seq library preparation

Flies taken from the −80°C storage were immediately immersed and homogenized in Qiazol (Qiagen), and the Qiagen RNeasy Universal Plus Mini kit was used to extract total RNA from whole body flies. RNA‐Seq libraries were prepared with the TruSeq stranded mRNA Library Prep Kit on a Neoprep device (software version 1.1.0.8 and protocol version 1.1.7.6, Illumina) starting with 100 ng of total RNA and using the default settings for an insert size of 200 bp and 15 PCR (polymerase chain reaction) cycles. We avoided batch effects by randomizing all libraries across library cards with identical lot number. Reads of 50 bp were sequenced on the Illumina HiSeq 2500 platform.

### 2.4 RNA‐Seq data analysis

Sequenced reads were trimmed with readtools (version: 1.5.2) (Gómez‐Sánchez & Schlötterer, 2018) based on a quality score of 20 and mapped to the *D. melanogaster* reference genome (version 6.03) (Hoskins et al., 2015) using gsnap (version: 2018‐03‐25) (Wu & Nacu, 2010) with the following parameters (‐A: SAM, ‐k: 15, ‐N: 1, ‐m: 0.08). Quality checks for even gene body coverage with rseqc (Wang et al., 2012) were used to exclude 3′‐biased libraries from the analysis. To quantify the number of exon‐aligned reads, we used rsubread (version: 1.30.9) (Liao et al., 2013) based on the annotation (version 6.09) of the *D. melanogaster* genome (Hoskins et al., 2015). Differential expression (DE) analysis was performed with edger (version: 3.22.5) (Robinson et al., 2010) between evolved (hot and cold) and ancestral replicates. To avoid biased analyses, we filtered lowly expressed genes by keeping only genes with a minimum 1 count per million reads in at least three samples.

We modelled the gene expression as following: *y* = Evolution + ℇ (*y* is the normalized expression level of each gene, and Evolution has three states: hot, cold and ancestral). Contrasts were made (i) between the average responses of hot‐ and cold‐evolved replicates to their common ancestors (contrast: *concordant evolution*) and (ii) between the evolutionary responses in hot‐ and cold‐evolved replicates (contrast: *divergent evolution*) similar to a recent study using the same experimental framework in *D. simulans* (Jakšić et al., 2019). The *p*‐values were adjusted for multiple testing according to Benjamini and Hochberg's false discovery rate (FDR) correction (Benjamini & Hochberg, 1995). Significant DE genes were further classified into groups of genes that exhibited distinct adaptive patterns based on the criteria shown in Table 1. The availability of the ancestral population provides the opportunity to polarize the gene expression changes in hot‐ and cold‐evolved populations (log‐scaled fold change in expression, log_2_FC). Although conceptually straightforward, separate contrasts between the hot‐ or cold‐evolved populations to the reconstituted ancestral population were not included in our main analysis because then the statistical inference for concordant and divergent evolution would rely on the intersection of two different tests, which leads to lower power in the identification of genes of interest.

**Table 1:** Statistical criteria and the numbers of genes with distinct evolutionary patterns

	Genes of interest	
	Laboratory adaptive (LA)	Temperature adaptive (TA)
Contrast: concordant evolution	Sig. (FDR < 0.05)	n.s.
Contrast: divergent evolution	n.s.	Sig. (FDR < 0.05)
Number of genes	541	203

### 2.5 Weighted gene co‐expression network analysis

Because gene‐wise differential expression analysis does not account for the nonindependence among functionally related genes it suffers from a lack of power, which is exacerbated by the required multiple testing corrections. Aiming for more statistical power, we performed a co‐expression analysis to identify gene modules that were coregulated during the temperature adaptation. Normalized gene expression (log‐scaled counts per million) of all genes were subjected to weighted gene co‐expression network analysis (WGCNA) implemented in the R package wgcna (Langfelder & Horvath, 2008). Briefly, Pearson's correlation coefficients were used to measure the co‐expression between each gene. Based on them we generated an adjacency matrix by raising the correlation coefficients with a power of β. A topological distance matrix was then calculated from the adjacency matrix and used for the hierarchical clustering to construct the network. We followed the developers' instructions and determined a β of 6 for our data and used the *blockwiseModule* function to construct a “signed” co‐expression network with a minimum module size of 100. For all others parameters we used default values. To determine the evolutionary pattern of each module, we investigated the normalized mean expression pattern of the genes in each module and tested whether they are enriched for adaptive genes.

### 2.6 Gene ontology enrichment analysis

To explore the broader biological context of the coregulated modules/genes with an evolved expression pattern, GO enrichment analysis was performed with the topgo package (Alexa et al., 2006). The “Weighted01” algorithm which accounts for the GO hierarchy was applied.

### 2.7 Comparison to published data sets

We compared the results of our experimental evolution study to other, published data sets to evaluate to what extent the patterns seen in our study can be generalized. Given the specificity of each study, we relied on the lists of candidate genes identified in the respective study.

Concordantly evolving genes in this study were compared to the candidate genes from an experimental evolution contrasting evolutionary responses in fluctuating and constant environments (Manenti et al., 2018) using Fisher's exact test for nonrandom association. The list of candidate genes was obtained from Sørensen (Table S1).

Genes/modules with a temperature adaptive response in this study (i.e., gene expression divergence in cold‐ and hot‐adapted laboratory populations) were compared to genes differing in natural populations from high and low latitudes (Hutter et al., 2008; Zhao et al., 2015). Spearman's correlation test was applied to test the overall concordance of expression difference between hot‐ and cold‐evolved populations in the laboratory and in nature. We used Fisher's exact test for nonrandom association between candidate genes with statistically significant expression difference. Specifically, we used data from Hutter et al. (2008), who compared the gene expression differences between African and European *D. melanogaster* populations: additional file 1 for the expression of all genes and additional file 4 for candidate genes. From Zhao et al. (2015), who compared populations of two *Drosophila* species, we used *D. melanogaster* data from the 29°C common garden, while the expression difference of all genes was obtained by personal communication from Zhao (Table S2).

## 3 RESULTS

### 3.1 Evolution of gene expression in novel temperature environments

**Figure 2:**
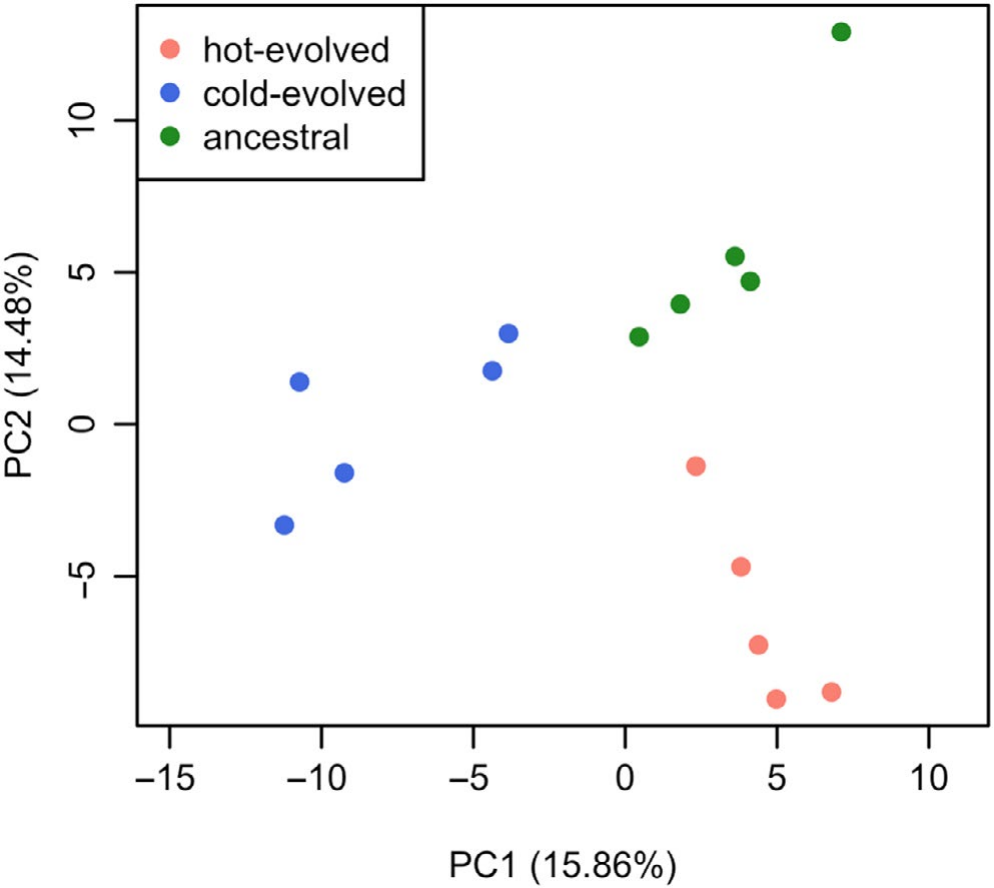
Transcriptomic divergence during adaptation to novel temperature regimes. Scatter plot showing the first and second principal components of the gene expression values in five replicates of three populations with different selection histories. Fill colours denote the evolutionary states of each sample. The principal component analysis demonstrates a clear transcriptomic divergence after adaptation to the novel temperature regimes

We measured gene expression in five replicates of hot‐evolved, cold‐evolved and reconstituted ancestral populations. After adapting for up to 150 generations to the novel temperature regimes, the transcriptome of populations from both treatments diverged significantly from their ancestors—probably an adaptive response to the new environments. Gene expression evolution can be visualized in a principal component analysis on the total transcriptomic variation. The first two PCs explained ~30% of the total variance. PC1 accounted for 15.86% of the variance and distinguished the cold‐evolved replicates from the others. PC2 separated the hot‐evolved replicates from their ancestors and explained almost as much variance as the first PC (14.48%) (Figure 2).

**Figure 3:**
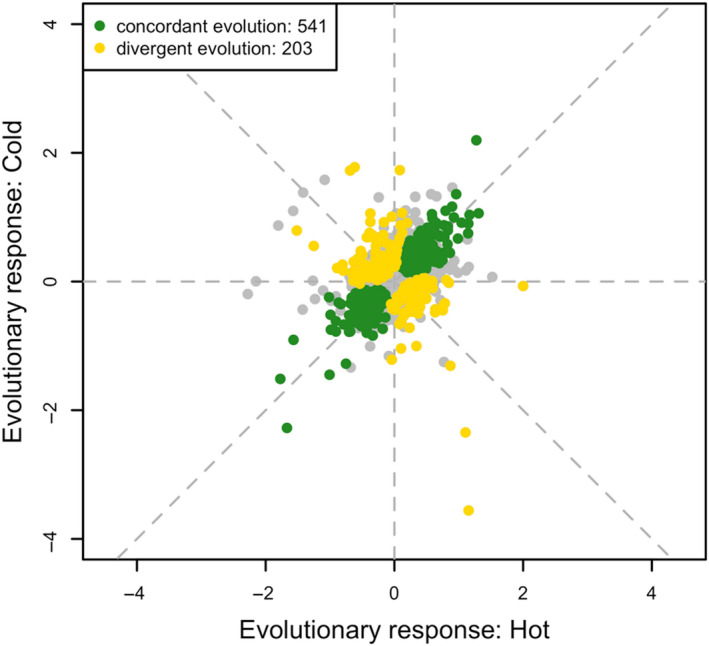
Genes exhibit distinct adaptive patterns under different temperature regimes. Scatter plot of expression changes (log_2_FC) comparing hot‐evolved replicates (*x*‐axis)/cold‐evolved replicates (*y*‐axis) to the reconstituted ancestors. Concordantly evolving genes (in green) fall on the diagonal where parallel changes can be found in both evolved populations. In contrast, genes that are specifically responding to one selection regime (in yellow) show inconsistent or opposing changes

Adaptation in the laboratory may involve the response to a common laboratory environment (e.g., daily temperature fluctuation, food and high rearing density) or to the specific experimental temperatures differentiating the two selection regimes (i.e., hot and cold environment). Genes contributing to the adaptation to the common environmental factors are expected to evolve consistent expression differences in both hot and cold replicates while genes affecting temperature adaptation would exhibit diverging expression changes in the two populations. We identified genes involved in adaptation with a linear model with two contrasts (Table 1 and Figure 3): (i) comparing the average response of both evolved populations to the reconstituted ancestral populations (Contrast: *concordant evolution*) and (ii) comparing the evolutionary responses in hot‐ and cold‐evolved populations (Contrast: *divergent evolution*).

The two contrasts identified 541 genes (297 up‐regulated and 244 down‐regulated, Table S3) that changed their expression in the same direction in the evolved populations, independent of the temperature regime (Figure 3, concordant evolution). In total, 203 genes (Table S4) were significantly differentially expressed between populations from the two temperature regimes (Figure 3, divergent evolution). Seventy‐nine of these genes were expressed at higher levels in hot‐evolved populations while 124 genes were expressed more in cold‐evolved populations. For simplicity, we will call these candidate genes temperature‐adaptive genes throughout although we provide no empirical evidence that the expression changes directly affect temperature‐specific fitness.

Rather than acting in isolation, genes are usually organized in functional networks with complex interactions. Hence, in addition to differential expression analysis on the level of single genes, we reconstructed the gene regulatory network based on the co‐expression pattern across the studied populations. Using wgcna, we identified 20 gene modules that exhibit different evolutionary patterns (Figure S1; Table 2). Enrichment for biological processes (GO terms, Table S5) of the genes in each module confirmed that these modules are biologically meaningful. An enrichment analysis of the genes in each module relative to adaptive genes identified 10 modules that were significantly associated with different adaptive processes in our experiment (Figure S1; Table 2). For instance, 213 of the 244 genes consistently down‐regulated in both selection regimes were grouped into Module 2 (Fisher's exact test (FET), odds ratio = 68.66, *p* < 2.2 × 10^−16^). Around 1,000 additional genes with similar expression changes were identified in the same module. Although these genes were not statistically differentially expressed in the single gene analysis, their significant clustering suggests that they may also contribute to adaptation, similar to genes with a significant expression change. Two modules (Modules 4 and 16) were associated with up‐regulation of genes involved in adaptation to the culturing conditions other than mean temperature. Four modules (Modules 10, 12, 13 and 18) contained genes with higher expression in hot‐evolved populations, and three modules (Modules 5, 9 and 15) grouped genes that were more highly expressed in the cold‐evolved populations. Next, we used a GO enrichment analysis to explore the broader biological context of the genes/modules with an adaptive expression pattern (Tables S5 and S6).

**Table 2:** Coregulated gene modules and enrichment analysis of GO and candidate adaptive genes in laboratory and natural populations

Module ID	Number of genes	Top GO term	This study^a^	Zhao et al. (2015)^b^
Module 0	1,573	Regulation of transcription, DNA‐templated		
Module 1	2,059	Axon guidance		TA_CH
Module 2	1,271	Mitochondrial electron transport, NADH to ubiquinone	LA_down	
Module 3	1,005	Cilium movement involved in cell motility		
Module 4	812	Multicellular organism reproduction	LA_up	
Module 5	694	Peptidyl‐proline hydroxylation to 4‐hydroxy‐l‐proline	TA_CH	
Module 6	636	Cytoplasmic translation		
Module 7	553	Proteasome‐mediated ubiquitin‐dependent protein catabolic process		
Module 8	496	Multicellular organism reproduction		
Module 9	330	Mannose metabolic process	TA_CH	
Module 10	299	Thiosulfate transport	TA_HC	
Module 11	296	Fatty acid elongation, saturated fatty acid		
Module 12	280	Cellular response to heat	TA_HC	
Module 13	266	Double‐strand break repair via break‐induced replication	TA_HC	
Module 14	261	Protein localization to microtubule plus‐end		
Module 15	242	Anterograde synaptic vesicle transport	TA_CH	TA_CH
Module 16	229	Germ‐band shortening	LA_up	
Module 17	199	Regulation of double‐strand break repair via homologous recombination		
Module 18	140	Detection of chemical stimulus involved in sensory perception of smell	LA_up TA_HC	TA_HC
Module 19	136	Mitochondrial ribosome assembly		
Module 20	109	Mitotic spindle organization		

^a^ Candidate adaptive genes in this study. LA_up/LA_down: laboratory adaptive genes showing consistent up‐/down‐regulation for laboratory adaptation; TA_HC: temperature adaptive genes evolving for higher expression in hot regime than in cold regime; TA_CH: temperature adaptive genes evolving for higher expression in cold regime than in hot regime.

^b^ Candidate adaptive genes in Zhao et al. (2015). TA_HC: temperature adaptive genes evolving for higher expression in low‐latitude habitat than in high‐latitude habitat; TA_CH: temperature adaptive genes evolving for higher expression in high‐latitude habitat than in low‐latitude habitat.

### 3.2 Concordant gene expression changes relate to temperature fluctuation and space constrain

Based on the GO enrichment analysis (Table S6), genes related to multicellular organism reproduction (GO:0032504, Figure 4) and mating (GO:0007618) were up‐regulated in both hot‐ and cold‐evolved populations. Genes involved in energy metabolic processes including mitochondrial electron transport (GO:0006120, GO:0006122 and GO:0006123, Figure 4), ATP synthesis coupled proton transport (GO:0015986) and tricarboxylic acid cycle (GO:0006099) were consistently down‐regulated in the evolved populations. The same terms were enriched in laboratory adaptation‐associated modules (Table S5: Modules 2 and 4). The up‐regulation of reproduction genes may reflect the increased male competition due to the high population density in the limited space of vials/bottles, similar to most laboratory evolution experiments (Yun et al., 2018). The limited space in vials/bottles may also explain the down‐regulation of genes involved in energy metabolism in both hot‐ and cold‐evolved populations. Flying is extremely energy consuming and in nature flies need to be always ready to do so. This requires the provision of sufficient energy without delay. Assuming that the constant provision of energy is costly and the ability to fly is strongly limited by the small volume of the bottles during maintenance in the laboratory, it is conceivable that the provision of energy for flying is disfavoured in our experiments. In addition, the rapid daily temperature fluctuations (10°C) shared between the two experimental treatments may explain the parallel response. Some support for this hypothesis comes from fitness measurements of populations that evolved in hot and cold fluctuating environments. Independent of their evolution regimes, evolved populations were consistently fitter than the ancestral population in both fluctuating hot (18–28°C) or cold (10–20°C) assay conditions. At constant assay temperatures, hot‐evolved flies were fittest at high assay temperatures, while cold‐evolved ones performed best at low assay temperatures (Tobler et al., 2015). The basis of such fitness differences has been further elucidated by the characterization of genes responsible for the adaptation to daily fluctuating temperatures (Manenti et al., 2018). Indeed, the 541 genes evolving concordantly in both temperature regimes showed a significant overlap with the 204 candidate genes for the functional adjustment to daily temperature fluctuations from Manenti et al. (2018) (FET, odds ratio = 2.70, *p* < .001). This suggests that at least some of the concordant expression changes between hot‐ and cold‐evolved populations reflect the adaptation to temperature fluctuation.

**Figure 4:**
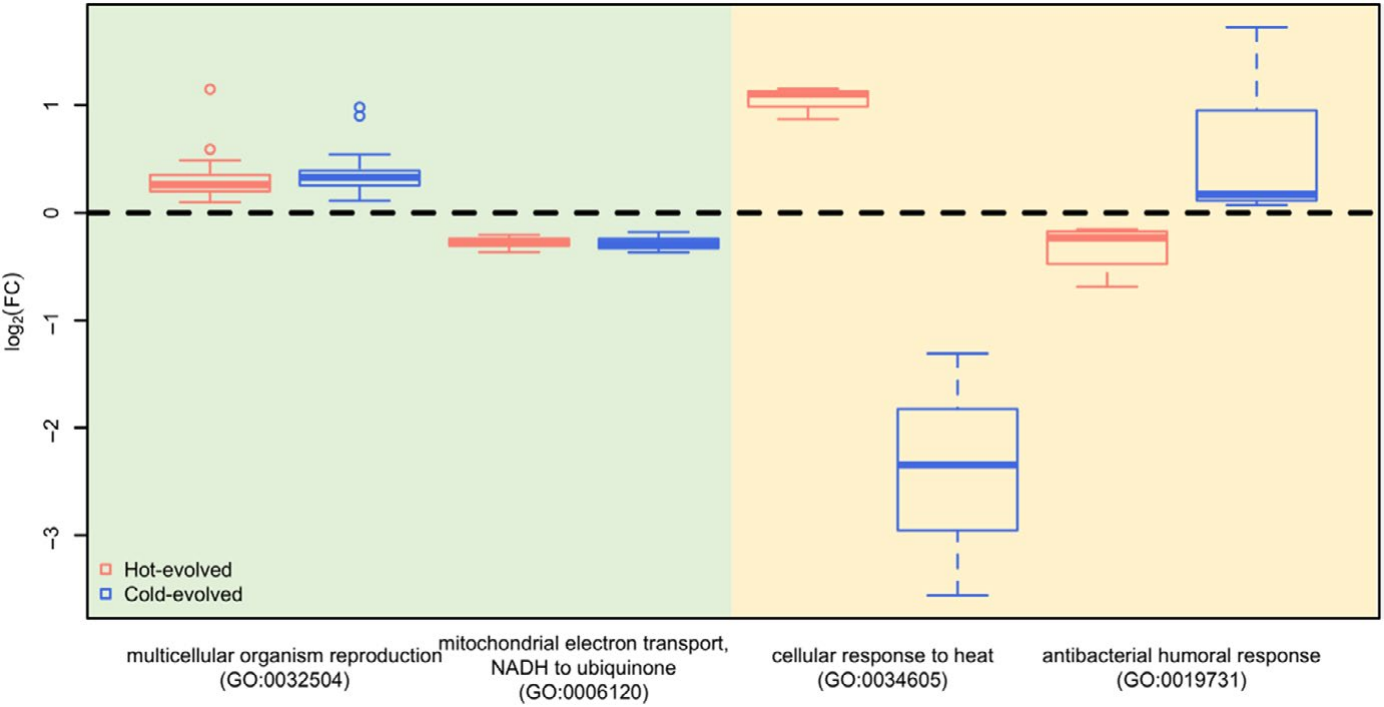
Biological processes involved in concordant and temperature‐specific adaptation. The concordant (green shading) and temperature‐adaptive (yellow shading) evolutionary response (log_2_FC in comparison to the ancestral populations) of genes enriched for different biological processes (red: hot‐evolved; blue: cold‐evolved). The tests can be found in Table S6 [Colour figure can be viewed at wileyonlinelibrary.com]

### 3.3 Temperature adaptation modulates cellular responses to environmental stresses

The 79 genes which had higher expression levels in hot‐evolved than in cold‐evolved flies showed significant enrichment for cellular response to heat (GO:0034605, Figure 4) and other abiotic environmental stimuli (Table S6). A module associated with this adaptive expression pattern (Module 12) showed the same enrichment pattern for cellular response to heat (Table S5). Remarkably, four of the five most divergent genes (based on fold‐change) evolving a higher expression in the hot temperature regime were *TotA*, *TotC*, *TotM* and *TotX* (all identified in Module 12), which represent half of the *Turandot* gene family. *Turandot* genes are involved in the cellular response to multiple environmental stressors, including heat and oxidative stress (Ekengren & Hultmark, 2001). It is possible that populations adapting to a fluctuating high temperature regime would benefit from a prepared cellular condition for regularly encountered temperature stress as discussed by Manenti et al. (2018). Interestingly, rather than a general response to temperature fluctuation, our result suggests that the up‐regulation of *Turandot* genes is specific to temperatures fluctuating around a high mean (23°C in our experiment). In addition to the responses to abiotic stimuli, we also identified significant enrichment of genes involved in the immune response at both single gene and module levels (Table S6 and Table S5: Module 18) (e.g., response to bacterium [GO:0009617] and proteolysis [GO:0006508]). Interestingly, the 124 genes and three modules (Modules 5, 9 and 15) with higher expression in cold‐evolved populations were enriched for similar biological processes related to immune responses (Tables S5 and S6), such as antibacterial humoral responses (GO:0019731, Figure 4) and regulation of antibacterial peptide biosynthetic process (GO:0002808). We hypothesize that this may reflect the co‐evolution of flies and microbes in novel temperature regimes. This hypothesis could be supported by the co‐evolution of ectothermic populations and their symbiotic microbes under different temperatures (Kokou et al., 2018).

### 3.4 Temperature‐induced gene expression evolution reflects temperature adaptation in natural populations

A key question for the interpretation of experimental evolution studies is how the results from the laboratory relate to adaptation processes in nature. We evaluated this by comparing genes with gene expression divergence in cold‐ and hot‐adapted laboratory populations to those identified by contrasting natural populations from high and low latitudes (Hutter et al., 2008; Zhao et al., 2015). Genes with higher expression in populations from low‐latitude habitats (i.e., Africa in Hutter et al., 2008 and Panama in Zhao et al., 2015) were significantly enriched among the 79 genes with increased expression in hot‐evolved replicates (FET, odds ratio = 3.90 and 3.05; *p* < .1 and *p* < .05). However, for the genes with increased expression in temperate habitats, we found a weaker and nonsignificant enrichment among the 124 genes with increased expression in our cold‐evolved replicates (FET, odds ratio = 1.44 and 1.53; *p* = .51 and .15). Interestingly, when compared to temperature adaptive gene modules (Modules 5, 9, 10, 12, 13, 15 and 18), adaptive genes in natural populations were significantly enriched in two modules showing corresponding adaptive expression patterns (Modules 15 and 18 in Table 2). Considering all expressed genes jointly, we found a significant positive correlation in temperature‐induced expression divergence between this study and each of the two temperature clines in natural populations (Spearman's correlation test, ρ = .10 and .16, *p* < .001 in both tests). To highlight the concordance between experimental and natural populations, we measured the correlation between the two studies using natural populations (Spearman's correlation test, ρ = .16, *p* < .001) and found this to be similar to comparisons with laboratory‐evolved populations. These results suggest that selection pressure caused by temperature manipulation in the laboratory mimics the natural selection in distinct habitats and leads to a concordant divergence of the transcriptome of experimental and natural populations when they are exposed to similar environmental changes.

## 4 DISCUSSION

Our study demonstrated a substantial change of gene expression in replicated *Drosophila melanogaster* populations evolving in hot and cold temperature regimes. Of key importance for the identification of the genes that share a similar evolutionary response across the two temperature regimes was the availability of an estimate for the gene expression in the ancestral population. We used an ancestral population, which was reconstructed from isofemale lines that have been maintained at small population sizes since the start of the experimental evolution experiment. While this procedure does not bias the allele frequencies in the reconstructed population (Nouhaud et al., 2016), and adaptation in the isofemale lines is highly limited given the small population sizes, it is possible that new mutations occurred during maintenance of the lines. Nevertheless, because we used a large number of isofemale lines to reconstruct the founder population, new mutations will be at too low a frequency to have a measurable impact on the inferred gene expression pattern.

We showed that the magnitude of transcriptome evolution in hot environments is similar to that in cold environments (Figure 2). This differs from a similar study in *D. simulans*, which found fewer genes with expression changes in flies from cold‐evolved populations (Mallard et al., 2018). Furthermore, the expression evolution of energy‐related genes was restricted to hot‐evolved populations (Mallard et al., 2018), while we found this evolutionary response in both temperature regimes. The nature of these differences is not clear but is unlikely to be a species‐specific effect given the previously observed parallelism between clinal *D. melanogaster* and *D. simulans* populations (Zhao et al., 2015). An important difference, however, is that, on the one hand, the flies in Mallard et al.'s (2018) study evolved for only 39 generations (rather than 81 in this study) in the cold environment, which may have resulted in an insufficient frequency change of important regulatory genes. On the other hand, Mallard et al. (2018) studied virgin males, while this study used mated ones, and mating status has an important influence not only on females but also on males (Ellis & Carney, 2010; Fowler et al., 2019). Further experiments are required to resolve this discrepancy.

The comparison of hot‐ and cold‐evolved replicate populations provided us with a robust data set to identify temperature‐adaptive genes in an experimental evolution setting. We find strong evidence that gene expression changes in the laboratory are informative about adaptation processes in natural populations. First, temperature‐adaptive genes in the laboratory overlapped significantly with temperature‐adaptive genes identified in two natural temperature clines. Second, we showed that several coregulated gene modules contributing to temperature adaptation in the laboratory are also enriched for temperature‐adaptive genes in natural populations. Third, we detected a significant positive correlation of the full transcriptomic responses of this study with each of the two studies on natural clines. Notably, the agreement between the studies on two different natural clines was not stronger than a comparison between each of them to this study on laboratory populations. Although significant, all correlation coefficients were moderate, which probably reflects methodological differences, such as assay conditions and methods to measure gene expression. It will be extremely interesting to repeat the comparison of gene expression differences evolved in natural clines and experimental evolution with a consistent methodological framework. We anticipate that such analyses will be particularly informative to understand to what extent pleiotropy restricts the evolution of gene expression in natural populations, but not in experimental evolution studies.

An interesting comparison with our results is an E&R study of *Chironomus riparius* populations which shared the same genomic response despite having evolved under different temperature regimes (Pfenninger & Foucault, 2020). In this study, we also identified some gene expression changes common to both temperature regimes (Figure 3), highlighting the impact of laboratory environment on expression levels. Nevertheless, focusing on the differences between hot‐ and cold‐evolved populations, our analysis was more powerful to detect temperature‐specific responses than comparing hot‐ and cold‐evolved populations with the ancestors. It is not clear whether a more powerful design (more replicates and longer evolution) or analysis (divergence of populations evolved at different regimes) would have uncovered temperature‐specific responses in *C. riparius*. Nevertheless, with a predominant temperature‐specific genomic selection response in this experiment (Tobler et al., 2014) as well as in a *D. simulans* E&R study (Otte et al., 2020), we propose that laboratory environments have a different impact across species. The reason for this difference is not clear—the laboratory culturing conditions may have matched the natural conditions better for *Drosophila* than for *C. riparius*.

Although this evidence suggests that *Drosophila* could be well suited to extrapolate from adaptive responses in the laboratory to selection pressure in natural populations, we caution that this may be highly contingent on the choice of phenotypes. Earlier generations of the same *D. melanogaster* experiment did not conform to the expectations from natural populations for a range of high‐level phenotypes which are frequently associated with temperature adaptation (Tobler et al., 2015). Similarly, an experimental evolution study carefully matching the seasonal temperature variation of *D. melanogaster* along the Australian cline failed to replicate the phenotypic clines from natural populations in the laboratory (Kellermann et al., 2015). Because some of the high‐level phenotypes evaluated in both studies (e.g., heat knockdown and chill coma resistance) are measured at extreme temperatures, these phenotypes were less likely to be directly selected. Rather, they may serve as an integrated phenotypic readout of temperature adaptation. Nevertheless, there are other laboratory studies conforming to the expectations from natural populations (Cavicchi et al., 1995; Huey et al., 1991; Stazione et al., 2020). This inconclusive behaviour could be explained by the correlated response of high‐level phenotypes with the adaptive phenotypes which may or may not be broken in different laboratory experiments. We propose that the identification of the adaptive phenotypes that confer direct fitness increase in response in an altered temperature regime (see box 2 in Barghi et al., 2020) will provide a much more promising approach to understand the differences and similarities of temperature adaptation in the laboratory and the wild.

## AUTHOR CONTRIBUTIONS

C.S. and S.‐K.H. conceived the study. C.B. and S.‐K.H. performed the analysis. V.N. prepared all RNA‐Seq libraries and supervised the common garden experiments and the maintenance of the evolution experiment. S.‐K.H., C.B. and C.S. wrote the manuscript. All authors read and approved the final manuscript.

## Supporting information

Fig S1

Table S1‐S2

Table S3

Table S4

Table S5

Table S6

## Data Availability

Sequence reads from this study will be available from the European Sequence Read Archive (http://www.ebi.ac.uk/ena/) upon publication. Original data and scripts for the analysis are available upon publication on the GitHub repository of this study (https://github.com/ShengKaiHsu/Dmel_parallel_temperature_adaptation).
