# Supplementary figures and images for: Parallel gene expression evolution in natural and laboratory evolved populations
Source: Mol Ecol. 2020 Oct 12;30(4):884–94. doi: 10.1111/mec.15649 (PMC7891358; doi:10.1111/mec.15649)

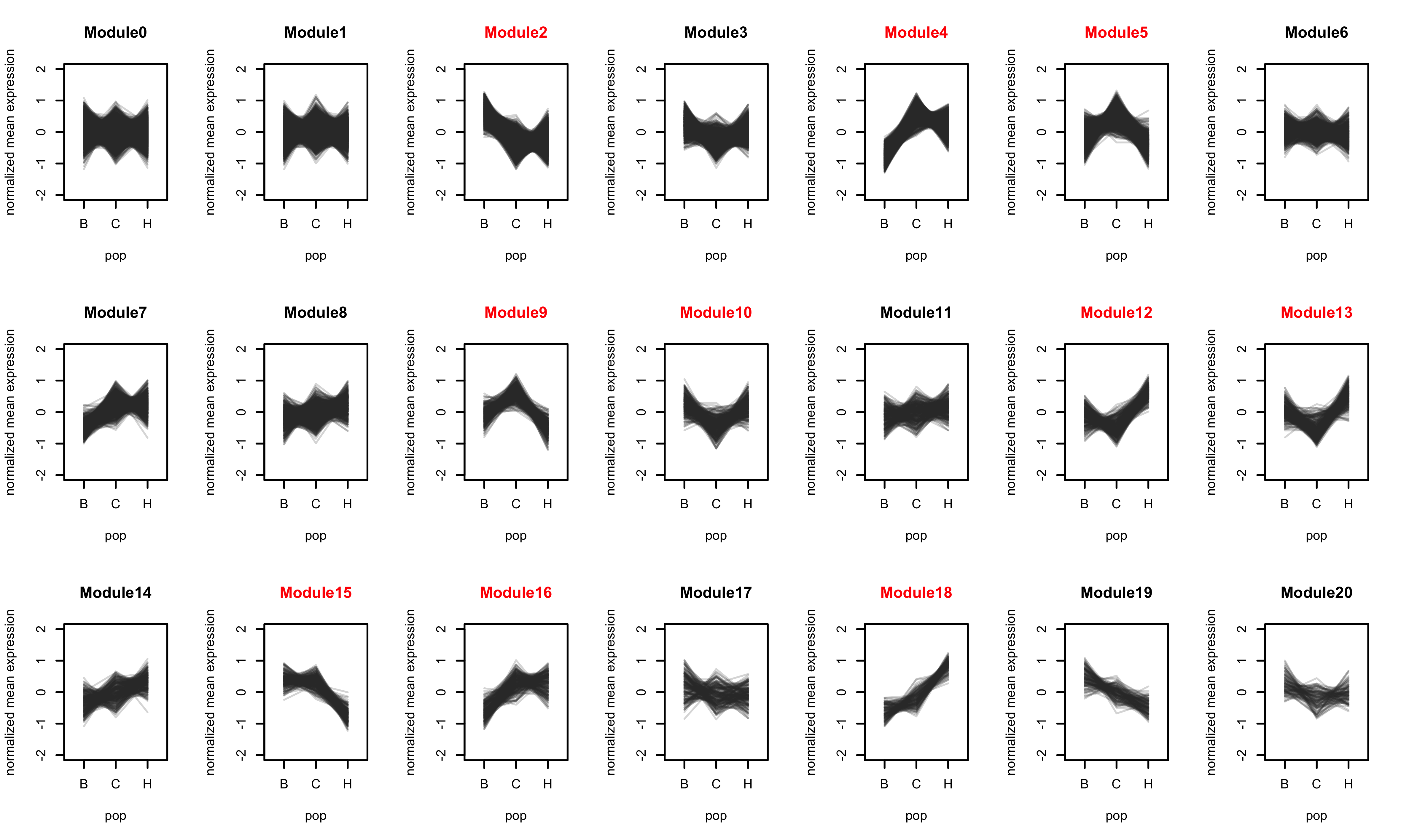

Supplement: Supplementary file 1 — Fig S1 [file MEC-30-884-s001.png]
